# The reproducibility of late gadolinium enhancement cardiovascular magnetic resonance imaging of post-ablation atrial scar: a cross-over study

**DOI:** 10.1186/s12968-018-0438-y

**Published:** 2018-03-19

**Authors:** Henry Chubb, Rashed Karim, Sébastien Roujol, Marta Nuñez-Garcia, Steven E. Williams, John Whitaker, James Harrison, Constantine Butakoff, Oscar Camara, Amedeo Chiribiri, Tobias Schaeffter, Matthew Wright, Mark O’Neill, Reza Razavi

**Affiliations:** 1 School of Biomedical Engineering and Imaging Sciences, King’s College London, St Thomas’ Hospital, Westminster Bridge Road, London, SE1 7EH UK; 2 PhySense, Department of Information and Communication Technologies Department, Universitat Pompeu Fabra, Barcelona, Spain; 3 Department of Cardiology, St Thomas’ Hospital, London, UK

**Keywords:** Atrial fibrillation, Cardiac magnetic resonance imaging, Catheter ablation, Atrium, Optimization, Late gadolinium enhancement

## Abstract

**Background:**

Cardiovascular magnetic resonance (CMR) imaging has been used to visualise post-ablation atrial scar (PAAS), generally employing a three-dimensional (3D) late gadolinium enhancement (LGE) technique. However the reproducibility of PAAS imaging has not been determined. This cross-over study is the first to investigate the reproducibility of the technique, crucial for both future research design and clinical implementation.

**Methods:**

Forty subjects undergoing first time ablation for atrial fibrillation (AF) had detailed CMR assessment of PAAS. Following baseline pre-ablation scan, two scans (separated by 48 h) were performed at three months post-ablation. Each scan session included 3D LGE acquisition at 10, 20 and 30 min post administration of gadolinium-based contrast agent (GBCA). Subjects were allocated at second scan post-ablation to identical imaging parameters (‘Repro’, *n* = 10), 3 T scanner (‘3 T’, n = 10), half-slice thickness (‘Half-slice’, n = 10) or half GBCA dose (‘Half-gad’, n = 10). PAAS was compared to baseline scar and then reproducibility was assessed for two measures of thresholded scar (% left atrial (LA) occupied by PAAS (%LA PAAS) and Pulmonary Vein Encirclement (PVE)), and then four measures of non-thresholded scar (point-by-point assessment of PAAS, four normalisation methods). Thresholded measures of PAAS were evaluated against procedural outcome (AF recurrence).

**Results:**

A total of 271 3D acquisitions (out of maximum 280, 96.7%) were acquired. At 20 and 30 min, inter-scan reproducibility was good to excellent (coefficient of variation at 20 min and 30 min: %LA PAAS 0.41 and 0.20; PVE 0.13 and 0.04 respectively for ‘Repro’ group). Changes in imaging parameters, especially reduced GBCA dose, reduced inter-scan reproducibility, but for most measures remained good to excellent (ICC for %LA PAAS 0.454–0.825, PVE 0.618–0.809 at 30 min). For non-thresholded scar, highest reproducibility was observed using blood pool z-score normalisation technique: inter-scan ICC 0.759 (absolute agreement, ‘Repro’ group). There was no significant relationship between indices of PAAS and AF recurrence.

**Conclusion:**

PAAS imaging is a reproducible finding. Imaging should be performed at least 20 min post-GBCA injection, and a blood pool z-score should be considered for normalisation of signal intensities. The clinical implications of these findings remain to be established in the absence of a simple correlation with arrhythmia outcome.

**Trial registration:**

United Kingdom National Research Ethics Service 08/H0802/68 – 30th September 2008.

**Electronic supplementary material:**

The online version of this article (10.1186/s12968-018-0438-y) contains supplementary material, which is available to authorized users.

## Background

The technique of three dimensional (3D) late gadolinium enhanced (LGE) cardiovascular magnetic resonance (CMR) imaging for the assessment of post-ablation atrial scar (PAAS) has been used for almost a decade [1, 2] but its reproducibility has never been formally quantified. An assessment of reproducibility is crucial from both a clinical and research perspective as the use of the technique becomes increasingly mainstream [3–8]. From a clinical perspective, confidence in the technique should be founded upon the knowledge that the location of PAAS remains fixed between scanning sessions. At a research level, the reproducibility of an imaging technique has a profound impact upon the design and scaling of research studies [9] and the interpretation of its results. This study aimed to quantify the reproducibility of imaging of PAAS, between multiple acquisitions, scanning sessions and established variations in imaging protocols.

There is no single established metric of PAAS imaging for comparison in assessment of reproducibility. Whilst some have looked to determine scar burden and location [7, 10, 11], others have sought to determine the presence of gaps in the ablation line [5, 6], each using bespoke thresholding and image interrogation techniques. Therefore, methods were developed specifically for this study in order to describe quantitative measures of PAAS imaging for reproducibility assessment: global PAAS burden (proportion of the left atrium (LA) occupied by PAAS, % LA PAAS), pulmonary vein encirclement (PVE- an objective method of quantification of ablation gaps), and a new method for point-by-point assessment of scar location .

Furthermore, recent studies of PAAS have tended to use very different scanning parameters. These variations include differing slice thickness (2–2.5 mm [3–5, 10] to 4 mm [6–8]), different gadolinium based contrast agents (GBCAs) and doses (varying from 0.1–0.4 mmol/kg of Multihance [10], Magnevist [7, 8], or Gadovist [5, 6]), and different scanner bore strengths (1.5 T [4, 6–8, 10], 3 T [5], or both [3]). Therefore, in assessment of reproducibility it was necessary to investigate the impact of these variations in detection and designation of PAAS. This cross-over study sought to determine the reproducibility of PAAS imaging within and across scanning techniques.

## Methods

### Study population

Between January 2014 and January 2016, all patients undergoing routine CMR imaging (Scan 0) prior to first-time atrial fibrillation (AF) ablation procedure were approached for participation. Forty subjects provided written and informed consent and the study was approved by the National Research Ethics Service (South London Research Ethics Committee reference 08/H0802/68). Exclusion criteria were contraindication to CMR imaging or prior allergic reaction to GBCA contrast. Baseline demographics and comorbidities were documented at the initial scan.

**Fig. 1:**
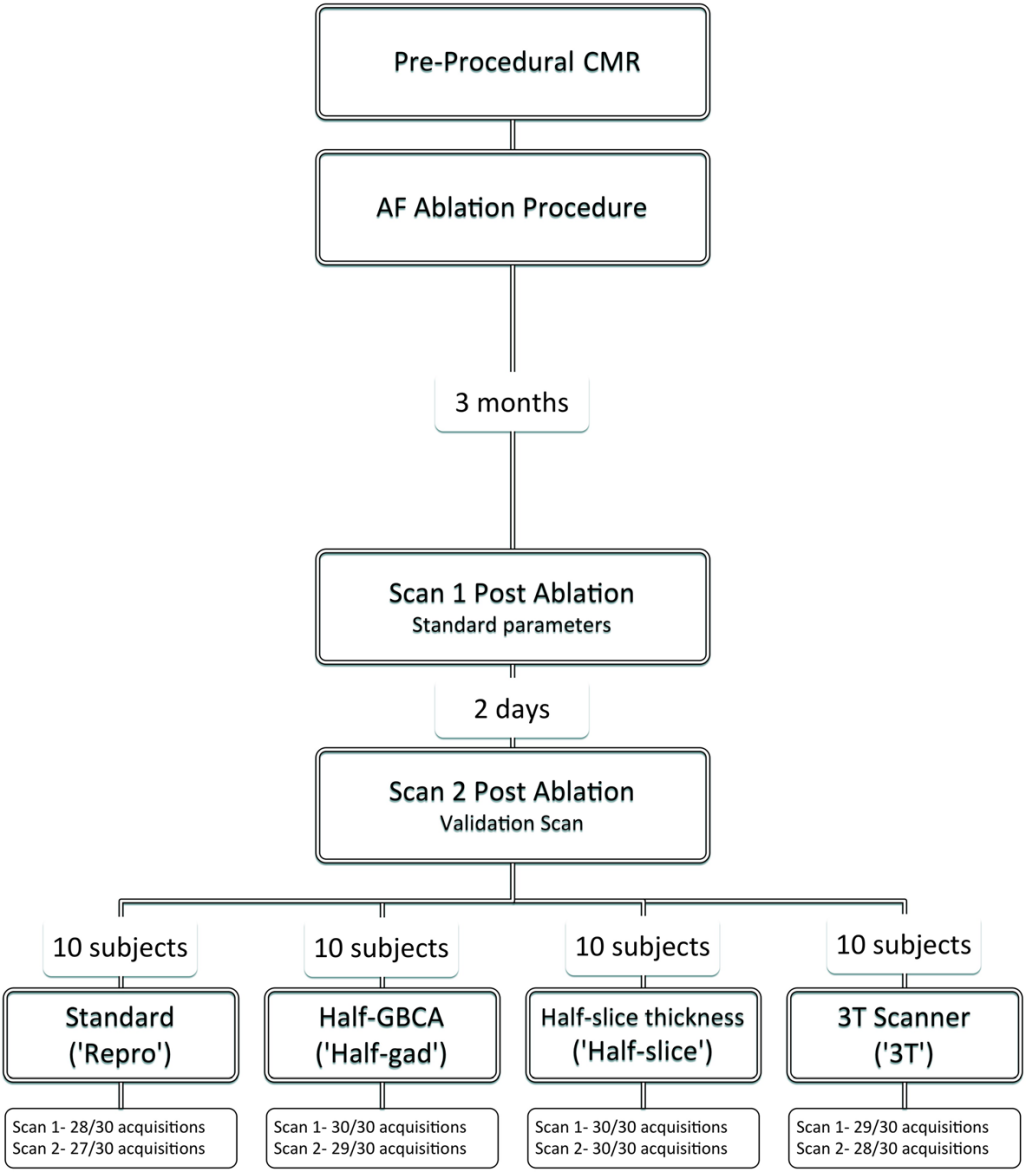
Flowchart demonstrating subject allocation and number of scan acquisitions achieved

All patients underwent further CMR imaging on two occasions following clinically indicated catheter ablation for AF (Fig. 1) The first post-ablation CMR scan (Scan 1) was performed at approximately three months after the ablation procedure, regardless of rhythm or arrhythmia recurrence (median 94 days, (interquartile range (IQR) 89–101 days)), and was performed using standard acquisition parameters (see below). A second scan session (Scan 2) was performed 2 days later (median 48.1 h, IQR 47.9–49.1 h). Subjects were allocated to scan 2 in 3 T scanner or the same 1.5 T scanner. 3 T scanner availability was limited, precluding randomisation of allocation, but the allocation (*n* = 10) was performed without reference to patient outcome or demographics. The remaining patients were randomised in equal ratios to one of three different imaging parameter groups for scan 2: repeat scan with identical acquisition parameters (‘Repro’ group, n = 10), repeat with half dose of GBCA (‘Half-gad’ group, n = 10), or repeat with half-slice thickness (‘Half-slice’ group, n = 10).

### CMR protocol

All CMR imaging was performed on a 1.5 T MR-scanner (Ingenia, Philips Healthcare, Best, Netherlands), except for those allocated to 3 T scanner for scan 2. All patients underwent detailed assessment at pre-procedural CMR scan (Scan 0), including left ventricular (LV) and right ventricular (RV) volumes and function, LA volumes and function, and 3D LGE assessment of baseline LA fibrosis. Details of the methods used to quantify baseline LA fibrosis are available in the online supplement. Cine imaging was performed in an end-expiration breathhold using a standard multislice balanced steady state free precession (bSSFP) technique (effective TR 2.7 msec, TE 1.3 msec, 1.4 × 1.4mm^2^ in-plane, slice thickness 10 mm, 50 phases). 3D inversion recovery spoiled gradient echo (LGE) acquisition was performed with coverage to include the whole of the LA in axial orientation. (TR 5.5 msec, TE 3.0 msec, flip angle 25°, low-high k-space ordering, respiratory and electrocardiogram (ECG)-triggering (end atrial diastole, maximum 120 msec acquisition window, respiratory navigator leading with gating window 5 mm), 1.3 × 1.3x4mm^3^ (typically 50 slices per acquisition, reconstructed to 0.94 × 0.94x2mm^3^), SPIR fat suppression, pixel bandwidth 540 Hz, phase-encoding direction anterior-posterior (AP), parallel imaging: SENSE P-reduction (AP) factor 2, 32 channel phased array digital receiver coil). Average acquisition window onset was 296 ± 40 msec post R-wave, and end at 398 ± 39 msec. GBCA dose for standard acquisition was 0.2 mmol/kg Gadovist (Bayer HealthCare Pharmaceuticals, Berlin, Germany). Respiratory gating artefact was minimised using an obtuse angulation of the navigator at the extreme right posterior aspect of the diaphragm, minimising excitation of pulmonary venous blood flow.

Scan 1 (post-procedure) was performed using the same 3D LGE acquisition parameters as the baseline scan, and a total of three LA 3D LGE datasets were acquired, timed to start at 10 min, 20 min and 30 min after GBCA administration. The inversion time was determined from a Look-Locker acquisition performed immediately prior to each LGE acquisition to ensure nulling of the myocardium. In rare cases in which the acquisitions took longer than 10 min, the subsequent acquisition was started immediately.

Scan 2 (post-procedure) was performed with specific modifications of the baseline scan, with acquisitions again performed at 10 min, 20 min and 30 min post GBCA administration.

1. **Reproducibility (‘Repro’):** *n* = 10, identical parameters to Scan 1.
2. **Half-gadolinium dose (‘Half-gad’):** n = 10, 0.1 mmol/kg of Gadovist
3. **Half-slice thickness (‘Half-slice’):** n = 10, the acquired voxel size was reduced to 1.3 × 1.3x2mm^3^ (reconstructed 0.625 × 0.625x1mm). Field of view remained unchanged to cover the entire LA, and therefore approximately 90–100 slices were acquired, doubling the nominal acquisition duration.
4. **3 T scanner (‘3 T’):** n = 10, scans were performed on 3 T scanner (Achieva, Philips Healthcare) with 32-channel coil. Parameters were matched to those for 1.5 T scanning as closely as possible (TR 4.0 msec, TE 2.0 msec, slice thickness 4 mm, pixel bandwidth 620 Hz, acquired voxel size 1.3 × 1.3x4mm^3^).

An ECG-triggered magnetic resonance angiogram (MRA) 3D dataset was also acquired at each scan as a high contrast template, delineating the LA endocardial border. The acquisition was commenced 90s after the start of a slow infusion of GBCA at 0.3 ml/s [12] (see online supplement for details), with the same coverage as the subsequent LGE acquisitions.

### Atrial fibrillation ablation protocol

Two experienced operators performed all catheter ablation procedures under general anaesthesia using Carto3 (Biosense Webster/Johnson&Johnson, Irvine, California, USA) electroanatomic mapping system, with the exception of 8 procedures performed using EnSite Velocity (St Jude Medical, St. Paul, Minnesota, USA). For patients with a diagnosis of paroxysmal AF and in sinus rhythm, a point-by-point wide area circumferential ablation (WACA) achieving pulmonary vein isolation (PVI) was performed using 8Fr irrigated SmartTouch catheter (Biosense Webster), or 8Fr irrigated TactiCath catheter (St Jude Medical). Target ablation parameters were > 5 g for at least 15 s per radiofrequency (RF) delivery location. Power was 30 W throughout except on the posterior wall, where it was limited to 25 W. Procedural endpoint was defined as PV isolation as confirmed on entry block (and exit block if capture achieved). For patients presenting with persistent AF, a WACA was performed followed by additional ablation lesion sets (mitral line, roof line, inferior posterior line, complex fractionated electrogram ablation) as a step-wise ablation.

### Imaging interrogation and comparison technique

For all subjects a semi-automated segmentation of the LA within the ECG-gated MRA acquisition was performed. The segmentation was then automatically registered (rigid registration with six degrees of freedom (3 translations and 3 rotations) [13]) independently to each LGE acquisition of the same imaging session (Acq_1_, Acq_2_, Acq_3_, see Table 1 for nomenclature). Mean translation was of magnitude 1.9 ± 1.6 mm, and rotation 0.62 ± 0.41°. For the subsequent imaging session at 48–72 h, the MRA acquisition at post-ablation scan 1 (MRA_1_) was automatically registered to the GMRA acquisition of post-ablation scan 2 (MRA_2_), which was itself then registered to each subsequent LGE acquisition (Acq_4_, Acq_5_, Acq_6_). Through this method, an identical atrial shell could be used for all six acquisitions for most subjects. The MRA was inadequate for semi-automated segmentation, according to visual assessment of poor contrast-to-noise ratio (CNR), in five subjects, for whom a manual segmentation of Acq_1_ was performed, and registered to all subsequent acquisitions.

**Table 1:** Image acquisition and comparison nomenclature

3D Late Gadolinium Enhanced Acquisition			Scan Session 1			Scan Session 2		
			10 min	20 min	30 min	10 min	20 min	30 min
			Acq_1_	Acq_2_	Acq_3_	Acq_4_	Acq_5_	Acq_6_
Baseline Scan	20 min	Acq_0_	C_0,1_	C_0,2_	C_0,3_	C_0,4_	C_0,5_	C_0,6_
Post ablation Scan 1	10 min	Acq _1_	–	C_1,2_	C_1,3_	C_1,4_	C_1,5_	C_1,6_
	20 min	Acq _2_	–	–	C_2,3_	C_2,4_	C_2,5_	C_2,6_
	30 min	Acq _3_	–	–	–	C_3,4_	C_3,5_	C_3,6_
Post-ablation Scan 2	10 min	Acq _4_	–	–	–	–	C_4,5_	C_4,6_
	20 min	Acq _5_	–	–	–	–	–	C_5,6_
	30 min	Acq _6_	–	–	–	–	–	–

Acq_i_ is the i^th^ post-ablation LGE acquisition for each subject, C_i,j_ is the comparison between Acq_i_ and Acq_j_

Second column is timing of commencement of acquisition, in minutes after administration of gadolinium based contrast agent

The CMR LGE volume was interrogated using a maximum intensity projection (MIP) technique, 1 mm inside endocardial shell and 3 mm beyond endocardial shell, and a single signal intensity (SI) value was assigned to each triangular face of the newly generated .vtk shell (typically 40,000 faces per LA shell). Comparison between two acquisitions *i* and *j* is termed C_i,j_ (see Table 1). Where only one subgroup was assessed, the comparisons are termed C_i,j_[repro], C_i,j_[half-gad], C_i,j_[half-slice] and C_i,j_[3 T] respectively. Multiway comparisons between acquisitions *i, j,... j + 1* are termed C_i,j…,j + 1_.

### Reproducibility of thresholded scar

Research groups have almost universally chosen to threshold PAAS, and a variety of normalisation methods and absolute thresholds have been implemented [4–8, 10, 14]. However, evidence for identification of thresholds has frequently relied upon correlation with voltage mapping techniques [14, 15], which are prone to registration and voltage sampling errors, or extrapolation from ventricular scar studies [16]. A histologically validated value of 3.3 standard deviations (SD) above the blood pool mean was therefore used for all indices where a single threshold value was required [17].

### Comparison of pre- and post-ablation atrial scar

In order to assess the impact of pre-ablation scar, a baseline analysis was performed to compare locations of scar on Acq_0_ (pre-ablation) to each of the post-ablation acquisitions. In contrast to the baseline fibrosis assessment, which was performed using a threshold of 0.97× blood pool SI on a mean intensity projection [15] (see Additional file 1), for this assessment Acq_0_ was instead thresholded at the post-ablation scar threshold of 3.3SD above blood pool mean on maximum intensity projection. Total proportion of the surface area of the Acq_0_ LA shell occupied by scar that would achieve the threshold for PAAS was assessed and compared to each post-ablation shell. A Sørensen Dice Similarity Coefficient (DSC) [18] was then calculated, assessing the co-location of pre- and post-ablation scar on a point-by-point basis (C_0,1_ to C_0,6_) (Eq. 1).

1 $$ {DSC}_{0,j}=\frac{2\left({Scar}_0\cap {Scar}_j\right)}{All\ {Scar}_0+ All\ {Scar}_j} $$

where DSC_0,j_ is the DSC for the comparison of Acq_0_ with Acq_j_, both thresholded at 3.3SD above the blood pool mean for the identification of scar.

To enable comparison where there was a change in LA shape or size following ablation, the pre-ablation shell was fused to the post-ablation shell using an iterative closest point method, blinded to scar location [8].

### Reproducibility of post-ablation thresholded scar

Two measures of thresholded PAAS were assessed. The first measure was that of the proportion of the surface area of the LA shell occupied by PAAS (%LA PAAS).

**Fig. 2:**
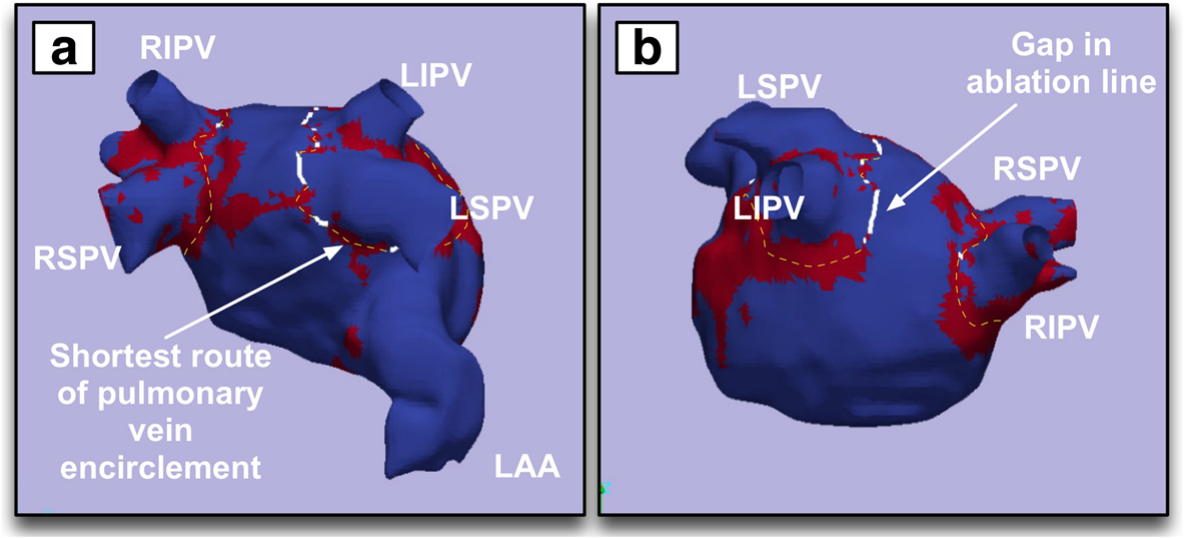
Illustration of derivation of pulmonary vein encirclement (PVE) measurement. The left atrial shell is thresholded at 3.3 standard deviations above the blood pool mean, with scar shown in red and healthy atrial myocardium in blue. **a** antero-superior view and **b** postero-lateral view of left atrium. The computed route of the pulmonary vein (PV) encirclement is shown in yellow dashed line, whilst detected gaps in the ablation line are shown in white. For this acquisition, the PV encirclement (PVE) for the right sided veins was 98.5%, and for the left was 81.5%. (LAA: left atrial appendage, LSPV: left superior PV, LIPV: left inferior PV, RSPV: right superior PV, RIPV: right inferior PV)

The second measure of PAAS was an objective measure of PVE by PAAS (a gap quantification method). In brief, the technique aimed to perform an objective measurement of the proportion of the WACA line that is occupied by uninterrupted scar on LGE CMR (Fig. 2) and the details of the derivation of PVE are documented in the online supplement.

### Reproducibility of scar imaging without thresholding

These two measures (%LA PAAS and PVE) are both highly dependent upon the scar threshold, and therefore the imaging reproducibility was also assessed point-by-point using a method that was not dependent upon thresholding. However, SI is expressed in arbitrary units, and therefore normalisation methods were required to enable inter-acquisition comparisons.

### Signal intensity normalisation methods

Shell surface SI units were normalised using four different acquisition specific reference methods, based upon established image analysis techniques [4–8]. Two of the methods are direct ratios, indexing by a single acquisition specific metric: blood pool image intensity ratio (BP-IIR- ratio of SI to blood pool mean) [4] and scar image intensity ratio (Scar-IIR- ratio of SI to best scar in reference slice) [5]. The other two methods index by both a mean and a variance: nulled myocardium z-score (V-Myo-Z: number of (ventricular myocardial) standard deviations (SD) from healthy ventricular septal myocardium mean) [7], and blood pool z-score (BP-Z: number of (blood pool) SDs from the blood pool mean) [6, 8, 14]. For example, the threshold used in this study (3.3 SDs above blood pool mean) is the equivalent of thresholding the BP-Z normalised shell at a value of + 3.3. Blood pool and scar reference values were obtained in a single standard slice at the level of the aortic root. For blood pool values, a 200mm^2^ circular ROI was placed in the LA blood pool, distant from potential artefact due to inflow enhanced by respiratory navigator signal; for atrial scar values, a 5mm^2^ ROI was placed within the most intense region of PAAS within slice. Ventricular myocardial mean and SD were obtained in the mid-septum in a region (50mm^2^) of homogenous signal intensity without blood pool contamination. Kurtosis and skew are not controlled for in any of the indexing systems.

### Shell comparisons

Following normalisation, intraclass correlation coefficients (ICCs) for both consistency and absolute agreement were calculated on a face-by-face basis (typically 40,000 triangular faces per shell), exploiting the identical morphology of the atrial shells.

### Association of post-ablation atrial scar with arrhythmia recurrence

Recurrence of AF post-ablation was defined as a recurrence of AF (>30s), or episodes of atrial tachycardia or atrial flutter, in line with HRS/EHRA guidelines [19]. Follow-up was at 3 months post-ablation, with symptom review, 24 h tape and 12-lead ECG performed. Subsequently, patients were typically reviewed at 6 and 12 months after the index procedure, and yearly thereafter. A 12-lead ECG ± holter monitor was performed at each review, in the absence of reported symptoms. If symptoms were reported, patients underwent 12-lead ECG, Holter monitor or event monitor assessment, according to symptom frequency. A blanking period of three months was employed post ablation and the %LA PAAS and %PVE were assessed against the binary outcome of recurrence of atrial arrhythmia. Where a repeat LA ablation procedure was performed, the presence or absence of electrical reconnection of each PV pair was recorded and corresponding PVE assessed.

### Statistics

Normally distributed continuous variables are presented as mean ± standard deviation, and median with interquartile range (IQR) for non-normal distribution or non-continuous ordinal data. Baseline characteristics and CMR indices were compared using χ^2^ test or Student t-test as appropriate. Statistics were analysed using SPSS Statistics (Version 22, International Business Machines, Armonk, New York, USA) unless otherwise stated. For the comparison of scales with intrinsic meaning and ratio scale, such as %LA PAAS or PVE, and where identical imaging parameters were used (‘Repro’ group), a within-subject coefficient of variation (WCV) was calculated using a root mean square method [20], otherwise an ICC was employed. ICC was calculated using a two-way mixed effects model on the assumption that the measurement technique (sequence timing, acquisition parameters and indexing technique) was a systematic source of variance [21]. ICC was generated for both consistency and absolute agreement using Matlab (Version R2015a, The Mathworks, Inc. Natick, Massachusetts, USA), and the ICC plugin (Arash Salarian, Version 1.2), C-1 and A-1 type analysis for consistency and absolute agreement respectively. Inter-scan ICC was calculated for C_2,3,5,6_ (four-way comparison between Acq_2,3,5,6_). ICC of 0.41 to 0.60 was interpreted to represent “moderate” agreement, 0.61 to 0.80 “good” agreement, and > 0.81 “excellent” agreement [22]. Repeated measures one-way ANOVA was used to assess differences in ICC between normalisation methods, with Tukey’s range test used to correct for multiple comparisons.

## Results

The subject characteristics and acquisitions achieved are summarised in Table 2, and there were no significant differences between subjects that underwent Scan 2 in 1.5 T or 3 T scanner. There were a total of 231 out of maximum possible 240 post-ablation acquisitions completed (96.2%). 40 acquisitions were completed at 10 min on scan 1 (Acq_1_), 40 Acq_2_, 37 Acq_3_, 39 Acq_4_, 39 Acq_5_ and 36 Acq_6_ (Fig. 1).

**Table 2:** Baseline demographics, as assessed at the initial scan prior to ablation procedure

	All Subjects (*n* = 40)	Scan 2 1.5 T (*n* = 30)	Scan 2 3 T (*n* = 10)	p-value
Male	31 (78%)	22 (73%)	9 (90%)	0.27
Paroxysmal AF	20 (50%)	17 (56%)	3 (30%)	0.14
CHA_2_DS_2_VASC Score	1 (IQR 0–2)	1 (IQR 0–2)	0 (IQR 0–1.5)	0.28
AF duration (years)	3.0 (IQR 2.1–5.3)	2.5 (IQR 1.9–5.0)	5.5 (IQR 2.6–12.5)	0.19
Significant Comorbidities	22 (56%)	16 (53%)	6 (60%)	0.71
Age (years)	61 ± 10	61 ± 8	61 ± 13	0.99
Weight (kg)	88 ± 17	88 ± 18	87 ± 12	0.77
Height (cm)	176 ± 7.1	176 ± 6.4	177 ± 9.3	0.60
BMI (kg/m^2^)	28.4 ± 5.3	28.7 ± 5.9	27.6 ± 3.1	0.48
HR at baseline scan (bpm)	61 ± 10	61 ± 8	61 ± 13	0.99
Sinus rhythm at baseline scan	25 (62.5%)	19 (63%)	7 (70%)	0.70
LV ejection fraction (%)	60 ± 10	62 ± 10	58 ± 11	0.41
LA size (ml)	121 ± 32	122 ± 37	119 ± 19	0.75
LA fibrosis at baseline (%)	36.0 ± 13.9	36.7 ± 15.1	33.9 ± 9.3	0.49
LA ejection fraction (%)	30 ± 18	29 ± 19	34 ± 12	0.41
LV native T_1_ time (msec)	988 ± 22	991 ± 24	985 ± 21	0.33

P-value is for comparison between patients that underwent scan 2 in 1.5 T versus 3 T scanners. *LA* left atrium, *LV* left ventricle, *BMI* body mass index, *HR* heart rate, *bpm* beats per minute. LA fibrosis was determined on manual segmentation of the left atrial wall, and thresholded at an image intensity ratio of 0.97 to the blood pool mean- see Online Supplement for details [20]

### Reproducibility of thresholded PAAS imaging

#### Comparison of pre- and post-ablation atrial scar

**Fig. 3:**
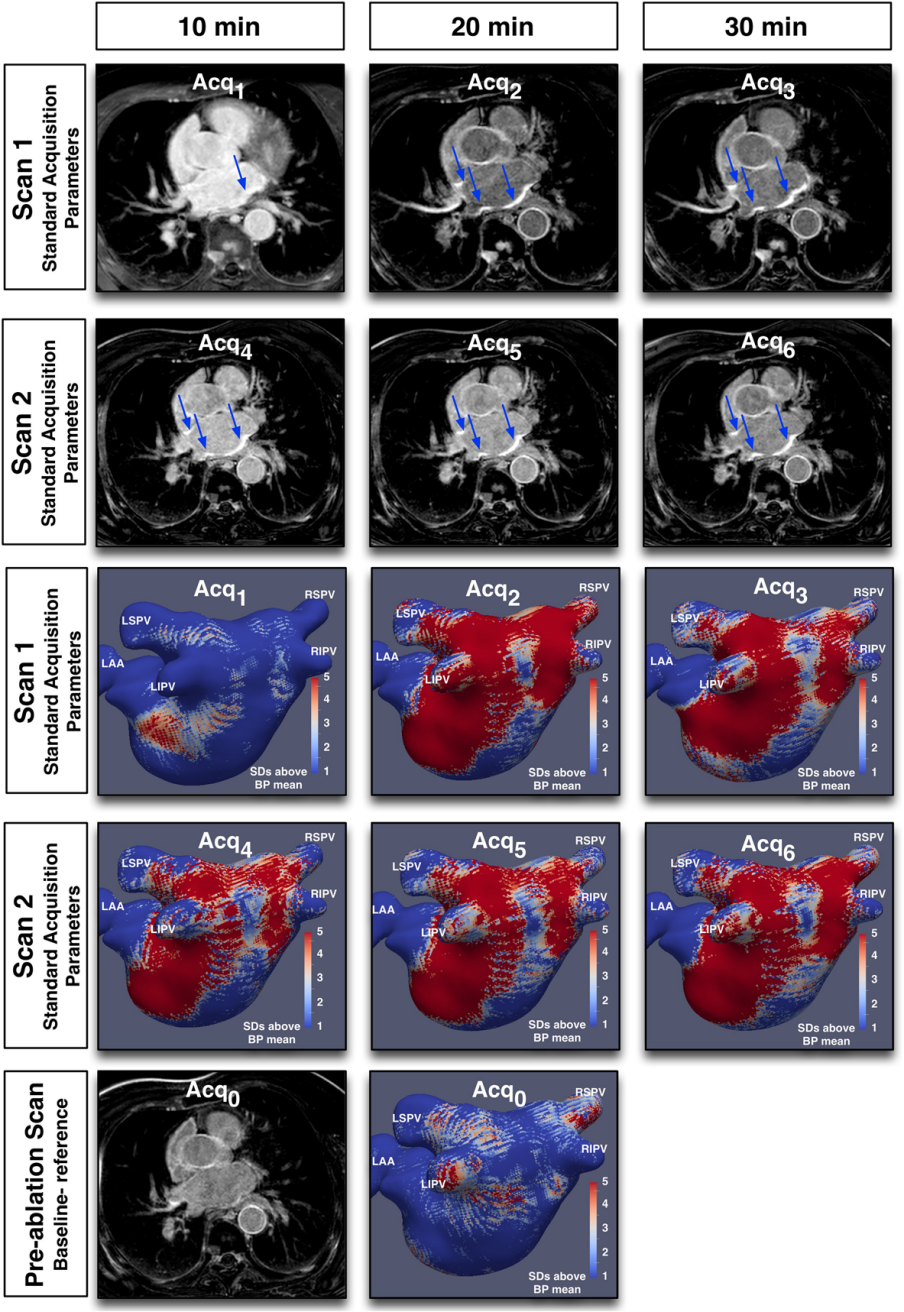
Examples of raw images and corresponding scar shells for a single subject. Scans 1 and 2 were performed using identical (standard) acquisition parameters, with acquisitions performed at 10 min, 20 min and 30 min post injection of gadolinium. Scan 2 was performed 2 days after Scan 1. Upper six panels show single representative slices of the 3D late gadolinium enhancement (LGE) dataset, at the level of the aortic root. The six panels below show corresponding scar shells, normalised according to blood pool z-score. Note the relatively poor reproducibility for acquisitions at 10 min, particularly Scan 1. The bottom two panels show the baseline scan, performed 20 min after gadolinium based contrast agent (GBCA) administration one month prior to ablation. Acq: acquisition. LSPV: left superior pulmonary vein, LIPV: left inferior pulmonary vein, RSPV: right superior pulmonary vein, RIPV: right inferior pulmonary vein, LAA: left atrial appendage, SD: standard deviation

When thresholded at the same value as PAAS (3.3 SD above the blood pool mean), a very small proportion of the LA shell was designated as baseline scar (median 0.62%, IQR 0.16–2.31%) (Fig. 3, bottom two panels). There was a very weak overall correlation between the proportion of pre-ablation scar and %LA PAAS (R^2^ = 0.024, *p* = 0.02 across all acquisitions). There was no significant correlation on assessment of each imaging parameter group (R^2^ for C_0,1_ to C_0,6_ ranged from 0.029 to 0.092, *p*-value 0.21 to 0.66). Overall average DSC, assessing co-location of pre- and post-ablation scar, across all acquisitions was very poor at 0.032 ± 0.009. Pre-ablation scar location was therefore interpreted to be unrelated to post ablation scar location and of minimal significance in further assessment, and was not included in further analysis.

#### Proportion of LA shell occupied by PAAS

%LA PAAS increased significantly with time from GBCA for all acquisitions groups. Mean %LA PAAS at 10 min (Acq_1_ and Acq_4_) was 7.1% ± 6.8%, increasing to 21.7 ± 15.6% at 20 min (Acq_2_ and Acq_5_) and 28.0 ± 16.1% at 30 min (Acq_3_ and Acq_6_) (*p* < 0.0001).

**Fig. 4:**
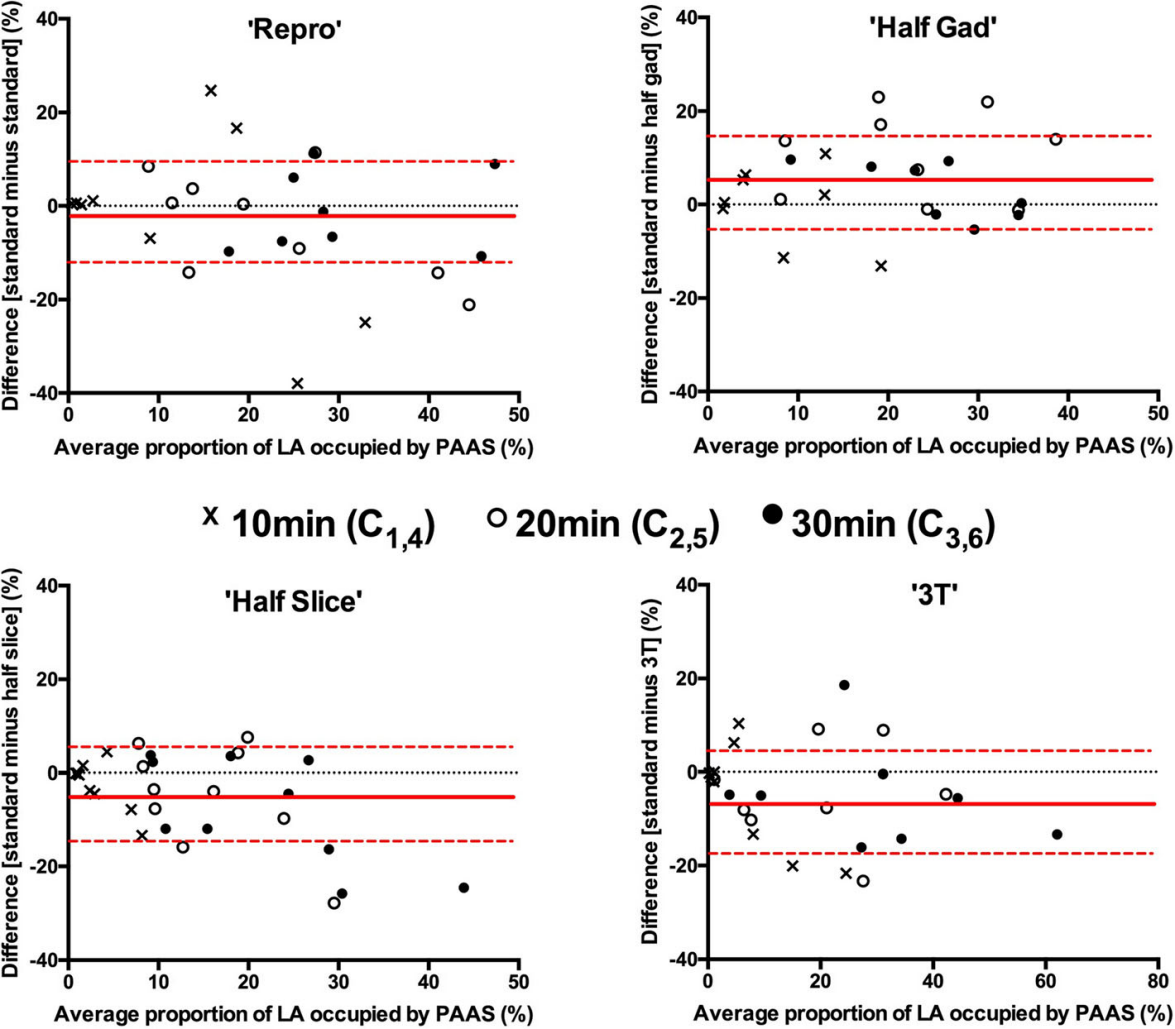
Bland-Altman plots demonstrating the reproducibility of the proportion of the LA shell occupied by post ablation atrial scar (PAAS), comparing scan acquisitions performed at the same time points post gadolinium administration. The top left chart shows the comparison for acquisitions performed with identical imaging parameters (‘Repro’). The other three charts show the reproducibility for those performed with differing imaging protocols, as previously detailed. Crosses show data points for comparison of acquisitions at 10 min post gadolinium (C_1,4_), open circles for comparison of acquisitions at 20 min post gadolinium (C_2,5_), and closed circles for comparison of acquisitions at 30 min post gadolinium (C_3,6_). Red lines show mean bias ±95% confidence interval

On interscan comparison of the acquisitions with identical imaging parameters (‘Repro’), reproducibility was poor at 10 min (C_1,4_- crosses in Fig. 4 top left panel) but improved markedly at 20 min (C_2,5_: open circles) and then 30 min (C_3,6_: closed circles). Reflecting this improvement, WCV was poor at 10 min (0.76) but improved significantly at 20 min (0.41) and again at 30 min (0.20) (*p* = 0.012).

Figure 4 also demonstrates Bland Altman plots where the different imaging parameters were used in Scans 1 and 2. Inter-scan ICCs (absolute agreement) generally improved from 10 to 20 to 30 min for each subgroup: for ‘Half-slice’ they were 0.070, 0.267 and 0.496, ‘Half-Gad’ 0.342, 0.485 and 0.454, and ‘3 T’ 0.020, 0.737 and 0.825 respectively. For comparison, the ICCs for the ‘Repro’ group were 0.182, 0.678 and 0.723 respectively.

Given the poor scar detection and the very low reproducibility at 10 min, Acq_1_ and Acq_4_ were excluded from further analysis.

#### Pulmonary vein encirclement

**Fig. 5:**
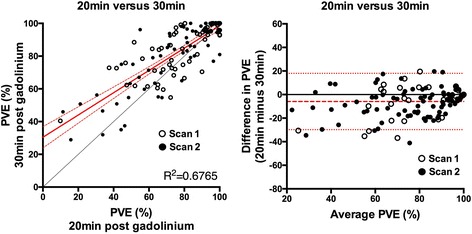
Impact of time from GBCA administration upon pulmonary vein encirclement (PVE)

PVE was significantly lower at 20 min than 30 min (76.4 ± 21.9% versus 82.3 ± 18.1%, *p* < 0.001): mean bias was − 5.9% (95% confidence interval − 29.8% to + 18.0%) (Fig. 5).

**Fig. 6:**
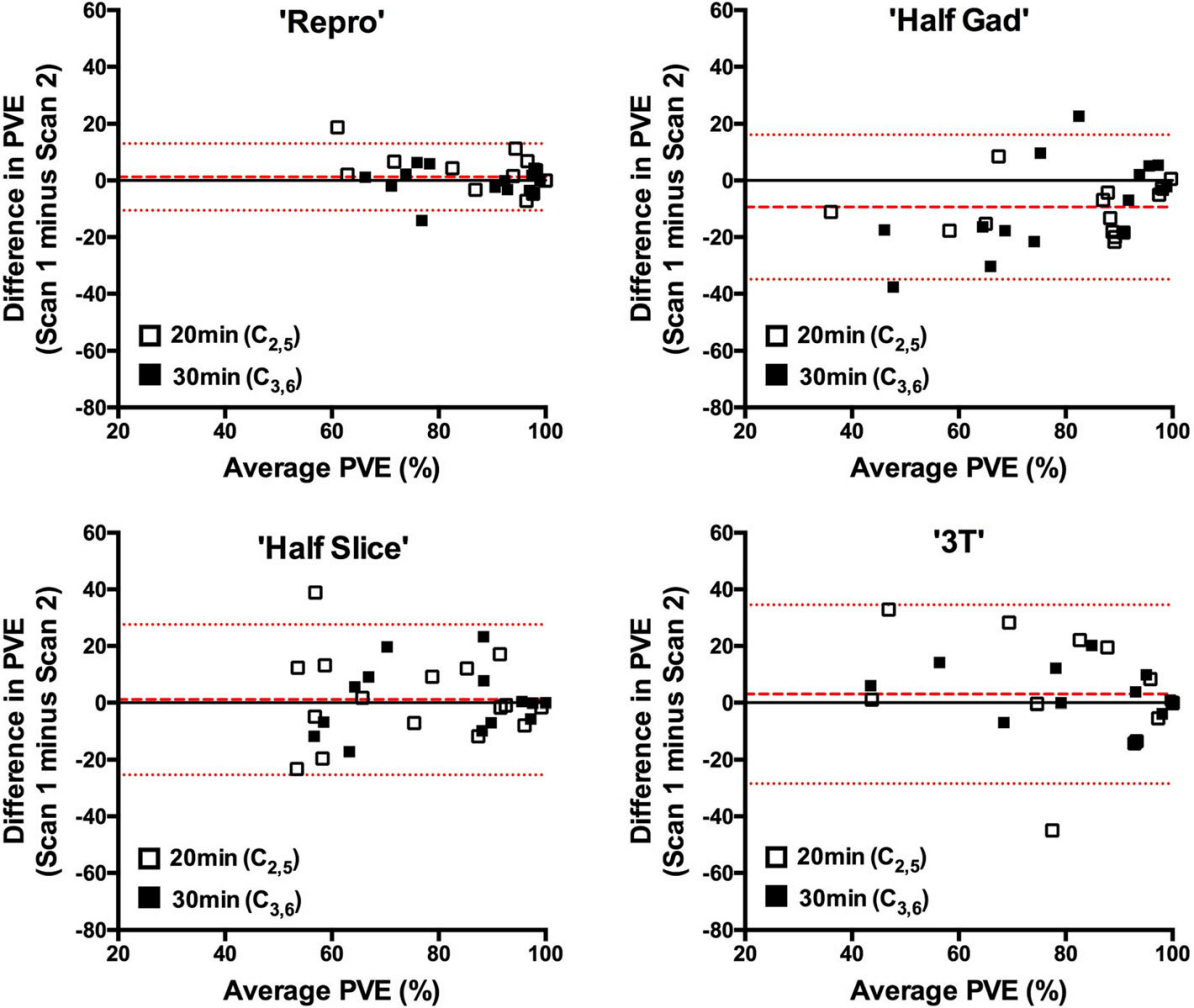
Reproducibility of pulmonary vein encirclement (PVE) measurements. Bland-Altman plots demonstrate reproducibility of measurements performed at 20 min post GBCA (C_2,5_ (open squares)) and 30 min post GBCA (C_3,6_ (closed squares)). Red lines show mean bias ±95% confidence interval

For inter-scan comparisons of the acquisitions with identical imaging parameters (‘Repro’), the WCV was 0.126 at 20 min (C_2,5_[Repro]), improving to 0.045 at 30 min (C_3,6_[Repro], *p* = 0.02) (top left panel Fig. 6), reflecting a high degree of reproducibility at both time points.

When different parameters were used for the second scan, the reproducibility remained acceptable. With a half dose of GBCA, PVE was significantly higher at each time point compared to standard imaging (standard: 70.7 ± 26.0% versus half gad: 84.7 ± 15.6%, p < 0.001), but there was no significant inter-scan difference in PVE for ‘Half Slice’ or ‘3 T’ (*p* = 0.65 and 0.35 respectively) (Fig. 6). Inter-scan ICCs (absolute agreement) at 20 and 30 min for the ‘Half-gad’ group were 0.458 and 0.618, for the ‘Half-slice’ group were 0.626 and 0.781 and for the ‘3 T’ group were 0.697 and 0.809 respectively. For comparison, the ICCs in the ‘Repro’ group were 0.774 and 0.876 respectively.

An analysis of additional determinants of PVE (%LA PAAS and scan quality) is presented in the online supplement.

### Reproducibility of scar imaging without thresholding

The intra-and inter-scan ICCs for consistency and agreement using all four normalisation methods are shown in Table 3. Consistency reflects the trend for the same regions in each acquisition to have higher normalised signal intensities, whilst absolute agreement is a more relevant measure of reproducibility if a fixed scar threshold value is to be selected.

**Table 3:** Intraclass correlation coefficients (ICCs) for point-by-point comparison, using each normalisation technique

		Intra-scan (C_2,3_ and C_5,6_)		Inter-scan (C_2,3,5,6_)		Inter-scan (‘Repro’ only)	
		Consistency	Absolute Agreement	Consistency	Absolute Agreement	Consistency	Absolute Agreement
Normalisation Method	BP Z-score	0.796 (IQR 0.729–0.848)	0.750 (IQR 0.667–0.827)	0.713 (IQR 0.659–0.764)	0.670 (IQR 0.589–0.720)	0.790 (IQR 0.767–0.799)	0.759 (IQR 0.739–0.768)
	V-Myo Z-score	0.754 (IQR 0.688–0.815)	0.499 (IQR 0.355–0.702)	0.677 (IQR 0.622–0.742)	0.363 (IQR 0.258–0.458)	0.748 (IQR 0.744–0.788)	0.436 (IQR 0.339–0.549)
	BP IIR	0.788 (IQR 0.723–0.837)	0.743 (IQR 0.644–0.805)	0.691 (IQR 0.655–0.722)	0.628 (IQR 0.530–0.677)	0.770 (IQR 0.664–0.799)	0.679 (IQR 0.622–0.744)
	Scar IIR	0.801 (IQR 0.752–0.852)	0.772 (IQR 0.647–0.813)	0.721 (IQR 0.682–0.774)	0.618 (IQR 0.491–0.694)	0.809 (IQR 0.773–0.828)	0.691 (IQR 0.576–0.744)

Values are median (with interquartile range (IQR)). *IIR* image intensity ratio, *BP* blood pool, *V-Myo* ventricular myocardium

For the assessment of scans performed using the same acquisition parameters (C_2,3,5,6_[Repro]), the consistency and agreement was good for most normalisation methods. However, of note the absolute agreement using V-Myo Z-score was poor (0.436, IQR 0.339–0.549)), reflecting poor absolute reproducibility of scar imaging when this method is used to normalise SIs.

When different inter-scan imaging parameters were used, the highest ICCs for absolute agreement were observed with blood pool z-score normalisation (*p* = 0.038, Tukey’s range test). With this normalisation method, the inter-scan ICC (absolute agreement) remained as high as 0.670 (IQR 0.589–0.720), despite using the different acquisition techniques.

There was also high consistency and absolute agreement between 20 min and 30 min acquisitions at the same imaging session (intra-scan ICC 0.754–0.801 across all normalisation measures (C_2,3_ and C_5,6_)), except when V-Myo Z-score was used where there was poor absolute agreement (ICC 0.499, IQR 0.355–0.702)).

### Recurrence of atrial arrhythmia

Follow-up time post ablation was for a median 417 days (IQR 285–628 days), and in total there were 13 patients (33%) with a recurrence of AF or tachycardia. Eleven patients elected to undergo a further ablation procedure, with two patients undergoing conservative management (one with a single episode of AF successfully treated with intravenous flecainide at 566 days post ablation, and the second with a single electrical cardioversion at 98 days, just outside of the blanking period, both with no subsequent recurrence).

**Fig. 7:**
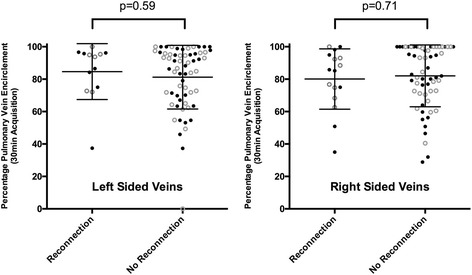
Relationship between percentage PVE and electrical reconnection. Electrical reconnection was assessed at repeat procedure in patients who had a sustained recurrence of arrhythmia (*n* = 11), and analysed as vein pairs (left and right, as indicated on charts). Closed circles indicate PVE at scan 1, open circles PVE at scan 2, and PVE scores are shown only for acquisitions at 20 min (Acq_2_ and Acq_5_) and 30 min (Acq_3_ and Acq_6_) post gadolinium

Overall %LA PAAS was 23.3 ± 14.2% in the no recurrence group, and 28.3 ± 20.2% in the recurrence group (*p* = 0.32). There was also no significant difference in average PVE between groups (no recurrence: 81.7% (IQR 63.2–96.3%), recurrence: 86.1% (IQR 73.2–95.4%), *p* = 0.10). Electrical reconnection of at least one PV pair was confirmed in 10 of the 11 subjects that underwent repeat ablation, and sites of reconnection versus respective PVE are shown in Fig. 7. There was no significant relationship between PVE and likelihood of electrical isolation of the vein pair: subjects could demonstrate very high or even complete PVE on CMR imaging, but still have electrical reconnection of the vein pair.

Complete PVE (> 99%) of both vein pairs was observed in a total of 11 acquisitions (out of total 152 ‘late’ acquisitions performed at 20 or 30 min post GBCA administration, 7%). Of these, a recurrence was observed on 3 occasions (27%, chi-square *p* = 0.94).

## Discussion

The key findings of this study can be summarised as follows:

1. There is good to excellent inter-scan reproducibility of thresholded PAAS imaging when identical imaging parameters are used
2. There is good inter-scan reproducibility of non-thresholded PAAS, provided that the signal intensities are normalised using appropriate methods
3. Reproducibility of PAAS imaging is better for acquisitions performed later (30 min) after GBCA injection
4. Reproducibility of PAAS imaging is significantly affected by the use of different imaging parameters, particularly different doses of GBCA, but remains acceptable when processed appropriately
5. There was no significant relationship between PAAS summary indices and AF recurrence

The reproducibility of the global summary indices of thresholded PAAS (%LA PAAS and PVE) was good to excellent for identical imaging acquisition parameters (‘Repro’), with WCV as good as 0.045 for imaging performed at 30 min post-GBCA administration. Such a finding suggests that the SD in PVE is less 5% of the PVE measurement, a high degree of consistency in the imaging of a structure as thin as the atrial wall, but the clinical implications of these small differences in measurements of PAAS remain to be established. These measures of reproducibility are of a similar magnitude to those of ventricular volume and function parameters (coefficient of variation (CV) 0.04–0.08 [23]) and ventricular scar imaging (CV 0.025–0.04 [24, 25]), both being widely accepted clinical imaging modalities. The reproducibility in this study compares favourably to that found for myocardial perfusion imaging (CV between 0.16 and 0.35) [23].

When non-identical imaging parameters were used, an ICC rather than WCV was used to assess for reproducibility in view of the alteration of the baseline observational technique. The ICCs for PVE reproducibility at 30 min remained ‘good’ (0.61 to 0.81) for all parameter alterations, and suggest that identical imaging parameters are ideal but not essential for comparison of PAAS imaging when imaging is processed appropriately. A change in the GBCA had the greatest impact upon reproducibility, with more PAAS detected, particularly at earlier timepoints post-GBCA administration. However, poor inter-scan reproducibility does not imply worse imaging with the altered parameter: reduced GBCA dose may improve PAAS detection and warrants further investigation, in line with recent ventricular scar LGE imaging studies [26].

Imaging quality was found to be poorer when the 3D LGE sequence was acquired at less than 20 min after GBCA administration, and this was reflected in very low markers of reproducibility for comparisons involving acquisitions at 10 min post-GBCA administration. A greater area of thresholded scar was consistently identified on scans acquired at 30 min than at 20 min. Moreover, on assessment of both %LA PAAS and PVE, the measurements were significantly more reproducible at 30 min, perhaps reflecting the higher CNR observed at this time point (Fig. 3) and the increased time for equilibration of contrast. PAAS identification, as opposed to ventricular scar identification, is critically dependent upon scar: blood pool contrast, rather than scar:healthy myocardium contrast and this is likely to explain the continued improvement in reproducibility at relatively late time points [26, 27].

The use of a single threshold (3.3 standard deviations above the blood pool mean) could have inappropriately strengthened or weakened the measures of reproducibility, depending upon the clinical accuracy of the threshold selected. It was therefore necessary also to employ a measure of reproducibility that was independent of thresholding, and the intra-scan and inter-scan reproducibility remained good. The ICC for inter-scan absolute agreement was as high as 0.759 on a point-by-point analysis when only the reproducibility group was assessed. This fell to 0.670 when all scanning parameter groups were assessed, but this still represents moderate to good reproducibility in the context of very different imaging parameters and no thresholding. These ICCs are for absolute agreement, which are important for comparison between scans, enabling the designation of alternative fixed thresholds. This study also suggests that it is valid to compare scans between patients and scanning sessions, even with different acquisition parameters, provided that the imaging is normalised or thresholded appropriately. Normalisation should ideally be performed using a blood pool Z-score, which was superior to all other methods on assessment of ICC for absolute agreement, but blood pool IIR and Scar IIR are only marginally inferior. The V-Myo Z-score normalisation demonstrated poor absolute ICC and should be avoided.

### Association of PAAS imaging and outcome

The absence of a significant relationship in this study between detection of gaps in the CMR-derived ablation line and recurrence questions the immediate relevance of PAAS imaging. This finding is in keeping with some recent studies [6, 28], but at odds with others which have demonstrated a significant relationship [5, 8, 10, 29]. However, it is important to review carefully those prior publications with positive findings, as they themselves have clearly delineated the limits of the relationship. For example, in one of the earliest studies of PAAS in 2009, Peters et al. found that the degree of scarring around only the RIPV was significant in predicting recurrence, thought to be likely to reflect the technical difficulty in isolating that vein and propensity of triggers to arise from that location [29]. Similarly, in 2010 Badger et al. found that only 10 out of 144 (7%) patients had complete scar encirclement of all PVs, but that there were no recurrences in this group, a statistically significant finding in a small subgroup [10]. The metrics used to assess gaps in this study are arguably more rigorous, but the overall proportion without a gap in both vein pairs’ encirclement is similar (7% of acquisitions had > 99% PVE of both veins), and in this case was not associated with recurrence.

However, the interplay of interruption of the continuity of the PVI lesion set and AF recurrence is a complex one: many gaps will not necessarily lead to recurrence of arrhythmia, whilst very small gaps may be sufficient for electrical reconnection. [30–32]. Further work is clearly required to fully understand the relationship between electrical and imaging gaps in scar post-ablation, and the impacts upon AF recurrence.

### Clinical implications

PAAS imaging is currently used primarily as a research tool, and enables the non-invasive evaluation of conventional and novel ablation therapies, including assessment of the impact of contact force [33], ablation-induced modification of fat pads containing ganglionated plexi [34], and also evaluation of ablation extent by cryoballoon [35]. The demonstration and quantification of the reproducibility of the imaging technique will facilitate the design and evaluation of further studies. Knowledge of the inter-scan variability assists in the determination of the sample size required to demonstrate a statistically significant alteration in the parameter assessed. Furthermore, this study suggests that sample size could also be reduced through ensuring that acquisitions are performed later (20 to 30 min) post GBCA administration, and, unsurprisingly, the use of identical imaging parameters. However, this study also suggests that a valid comparison of PAAS may be performed when it has not been possible to ensure identical imaging parameters [3], albeit with wider confidence intervals and the need for appropriate SI normalisation techniques.

The use of PAAS imaging to guide repeat ablation procedures is more controversial. The results of this study suggest that detection of PAAS is a reproducible finding, but the clinical implications for guidance of repeat procedures are unclear and warrant further investigation.

### Limitations

This study was performed at 3 months post ablation, and is an evaluation of chronic scar formation. As such, the results are not directly applicable to the assessment of acute lesion formation, and could not be used to guide acute repeat ablation during the index procedure in a hybrid-type environment. Likewise, there is evidence that there is a slow fading of scar with time [36], and the application of these results to imaging > 3 months post-ablation should be performed with caution.

There is also the possibility that the method of image interrogation could introduce bias towards improved reproducibility. The technique involved a rigid image registration step, in order to maintain morphologically identical LA shells which were important for subsequent assessments. The endocardial mask (GMRA acquisition) was generally registered to the subsequent LGE acquisitions (translation 1.9 ± 1.6 mm and rotation 0.62 ± 0.41°). For the majority of the subjects this was performed blinded to scar, using the GMRA sequence only. However, in five subjects the registration was of a 10 min acquisition to subsequent LGE acquisitions. The re-registration goodness of fit is evaluated across all high contrast features within the dataset, including bone and soft tissue, and therefore the effect of LA scar (approx 6 ml within 6000 ml dataset, < 0.1%) was felt to be negligible, particularly given the poor PAAS enhancement on the 10 min acquisitions. A further concern of the image processing technique, using a maximum intensity projection, is that adjacent bright structures such as the aorta may be interrogated. The processing technique, using an endocardial mask based upon the high contrast GMRA, aimed to minimise the contamination of signal from beyond the atrial wall, but there remains the possibility that reproducibility measures may have been increased by mis-interrogation of static structures.

The summary indices of PAAS were all developed specifically for this study and further validation of their robustness is warranted. In particular, the implementation of semi-automated steps in image interrogation has meant that inter- and intra-observer variability has not been explored in this study. Further evaluation of the variation in summary indices warrants the re-segmentation of the LA endocardium, with subsequent image processing steps repeated. Finally, there is no gold standard for assessment of accuracy of scar detection and delineation. Manual segmentation was considered, as has been performed in previous studies [11], but the inter- and intraobserver variability was high for this subjective measure. Core scar is clear to the expert observer, but the borderzone of the scar depends upon a user defined threshold and manual corroboration between slices: it is these areas that are of particular importance in the assessment of reproducibility but are inconsistent on manual segmentation.

## Conclusions

CMR imaging of PAAS is a reproducible finding when the 3D LGE dataset is acquired at least 20 min after the administration of GBCA. Inter-scan reproducibility is good to excellent when identical imaging parameters are used, and remains acceptable even when acquisition parameters differ significantly. The clinical implications of these findings remain to be established in the absence of a simple relationship between PAAS and AF recurrence.

## Additional file

Additional file 1: Reproducibility of Post-ablation atrial scar imaging- Supplementary Data. (DOCX 889 kb)

## Abbreviations

aCNR: apparent contrast-to-noise ratio
AF: atrial fibrillation
aSNR: apparent signal-to-noise ratio
BP-IIR: blood pool image intensity ratio
BP-Z: blood pool Z-score
bSSFP: balanced steady state free precession
CI: confidence interval
CMR: cardiovascular magnetic resonance
CNR: contrast-to-noise ratio
ECG: electrocardiogram
GBCA: gadolinium based contrast agent
GMRA: gated magnetic resonance angiogram
ICC: intraclass correlation coefficient
IQR: interquartile range
LA: left atrium/left atrial
LAA: left atrial appendage
LGE: late gadolinium enhancement
MRA: magnetic resonance angiogram
PAAS: post-ablation atrial scar
PV: pulmonary vein
PVE: pulmonary vein encirclement
PVI: pulmonary vein isolation
RF: radiofrequency
RV: right ventricle/right ventricular
SD: standard deviation
SI: signal intensity
TE: echo time
TR: repetition time
WACA: wide area circumferential ablation
WCV: within-subject coefficient of variation
