# Supplementary material for: The reproducibility of late gadolinium enhancement cardiovascular magnetic resonance imaging of post-ablation atrial scar: a cross-over study
Source: J Cardiovasc Magn Reson. 2018 Mar 19;20:21. doi: 10.1186/s12968-018-0438-y (PMC5858144; doi:10.1186/s12968-018-0438-y)
Supplement: Supplementary file 1 — Reproducibility of Post-ablation atrial scar imaging- Supplementary Data. (DOCX 889 kb) [file 12968_2018_438_MOESM1_ESM.docx]

# Reproducibility of Post-ablation atrial scar imaging- Supplementary Data

## Fibrosis Assessment

Atrial fibrosis analysis was performed on an MITK-based platform (German Cancer Research Centre, Heidelberg, Germany), with custom-build modifications to enable the quantification of atrial fibrosis. The LA endocardial surface was defined via manual segmentation within the 3D LGE volume on a slice by slice basis, using 3D interpolation to minimise slice-by-slice discontinuities. A 2mm surface dilation was used to define the epicardial border, in keeping with established methods [1], and a mean intensity projection technique through the defined atrial wall was used to ascribe a single signal intensity value to each point on the LA endocardial surface model (typically 20,000 polygons per shell). The mitral valve, distal pulmonary veins (2mm distal to antrum) and LA appendage were removed using the Clip filter in Paraview (Kitware, New York, NY, USA) and the surface was re-extracted as a binary file.

LA scar burden was quantified using an image intensity ratio threshold (0.97 times mean blood pool (BP) signal intensity [1]). BP signal intensity was measured for a 4ml spherical volume placed in the center of the LA blood pool, distant from artefact including respiratory navigator signal.

## Gated magnetic resonance angiogram acquisition

For post-ablation scans, a gated magnetic resonance angiogram (GMRA) 3D dataset was acquired as a high contrast template, delineating the LA endocardial border. The acquisition was commenced 90seconds after the start of a slow infusion of GBCA at 0.3ml/second [2]. 3D inversion recovery spoiled gradient echo acquisition with coverage identical to that of subsequent LGE acquisitions was used, to include the whole of the LA in axial orientation. TR 5.5msec, TE 3.0msec, flip angle 25°, low-high k-space ordering, increased receiver bandwidth (890Hz, fat-water ratio 0.5), respiratory and ECG gated (end atrial diastole, maximum 120msec window, identical to LGE acquisitions), 1.3x1.3x4mm^3^ with 2mm slice overlap (typically 50 slices per acquisition), SPIR fat suppression, empirical inversion time 200msec.

## Assessment of Imaging quality

Qualitative assessment of all acquisitions was performed independently by three experienced observers. Observers were presented with a single representative transverse slice at the level of the aortic root in random order, with 10 initial training sets, and 20 random acquisitions repeated in order to assess intra-observer reproducibility. Likert Scale assessment was performed, with acquisitions graded across four criteria: image sharpness, scar contrast, freedom from artefact and quality of myocardial nulling. All criteria were scored from 1-5, with a score of 5 indicating optimal imaging.

## Derivation of Pulmonary Vein Encirclement metric

The main stages of the method are:

- Left atrial (LA) segmentation and tissue characterisation.
- Semi-automatic labelling of regions within the left atrium
- Gap identification and quantification.

**1. Left atrial segmentation and tissue characterisation.**

LA segmentation was performed according to the methods described in the manuscript, and the resulting LA shell was thresholded at 3.3 standard deviations above the blood pool mean in order to define the locations of atrial scar.

**2. Semi-automatic labelling of the regions of the LA**

In order to determine consistent anatomical regions for each LA, a registration-based method was applied defining anatomical regions on a template atrium using previously described techniques [3]. An extra division in the middle of the atrium separating the left from the right side was also added, creating 28 regions in total, with particular attention paid to the regions surrounding the pulmonary veins (PVs) (Supplementary Figure 1).


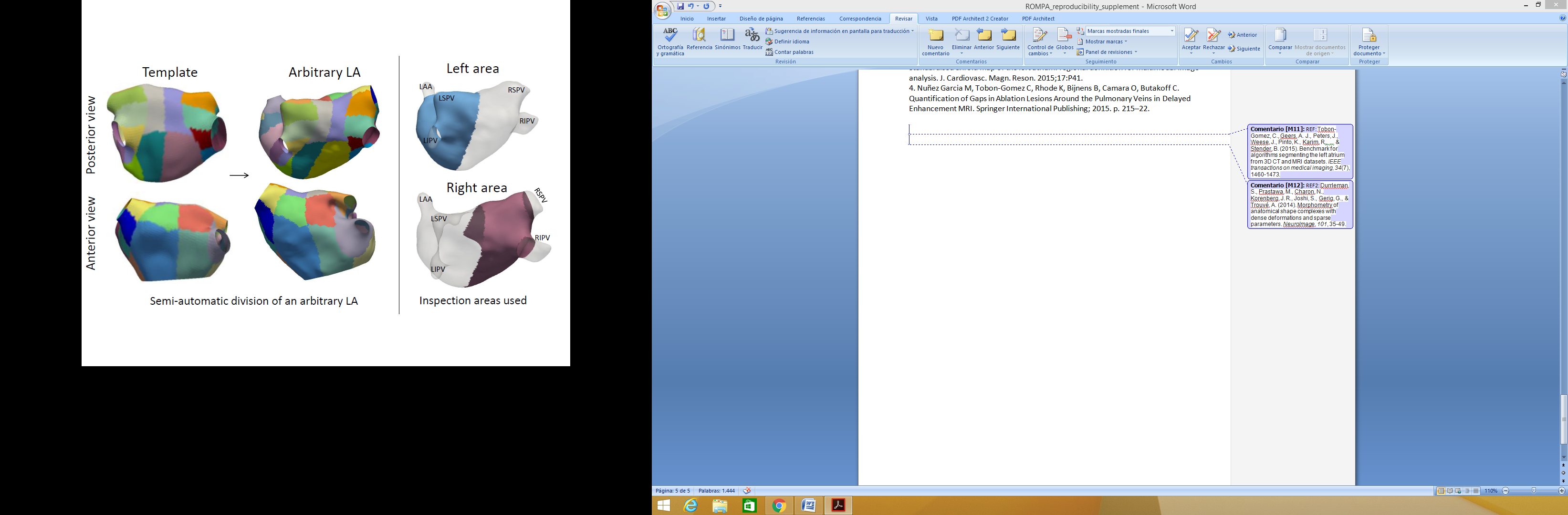


Additional file 1 Figure S1. Labelling of LA regions.

Left sided atrial shells demonstrate the standardised template and registration of the patient specific atrial shell. Right sided atrial shells show inspection areas used.

The uniform division of the LA was then transferred to each LA. First, the shells were standardised (semi-automated removal of PVs, left atrial appendage (LAA) and mitral valve (MV) orifice) following the process described by Tobon-Gomez and colleagues [4]. Five user-defined seeds were placed at the antrum of the PVs and the LAA, and lines connecting each PV (or the LAA) to the 2 PVs on the other side of the LA were calculated to define the lumen of the vein or appendage. The body of the atrium was then defined at the point along the connecting line where the contour of the vein (or the LAA) widened significantly, and an automated clipping was applied at 3mm from the LA body. Other clipping distances were trialed (0 to 10mm), with minimal difference in the final outcome observed. The MV was then cut automatically using the location of the placed seeds to define a suitable plane representing the MV position

Registration was then performed using an affine transformation followed by non-rigid registration using currents [5]. Region labels were transferred from the template to the registered mesh using the closest point approach and finally, the labels were transferred to the original (non registered) mesh where the gap analysis was performed. In this way, each vertex on the original (not template) LA shell was labelled according to region.

**3. Gap identification and quantification**

Gaps in the ablation line were quantified using a technique similar to that described in a previous publication by the Barcelona group [6]. The detailed labelling of the LA shell enabled the size of the wide area circumferential ablation to be defined accurately, and the veins were assessed in pairs. The ablation line was assessed at a relatively large maximum distance from the PV antrum (up to one quarter of the whole LA diameter) in order not to ignore appropriate ablation locations when a very wide ablation line was performed, isolating the veins in pairs. The isolating path was defined as the closed path that encircled the PV pair with the minimum gap length between regions of binarised scar along that pathway. A graph was constructed where each node represented a scar patch and the edges are the minimum distance between the corresponding patches. The Dijkstra algorithm was then applied to find the shortest path. Note that according to this method, healthy areas would only be defined as gap if they belonged to the isolating path.

In this way a Relative Gap Measure (RGM) was defined where:

$$RGM=\frac{Gap Length}{Total Length}$$

Where gap length is the sum of the length of all gaps in the isolating path and total length is the total length of the path (gaps + scar patches length).

As a more intuitive measure for clinical application, a Pulmonary Vein Encirclement (PVE) index was also defined where:

$$PVE=1-RGM$$

## Analysis of determinants of Pulmonary Vein Encirclement

Supplementary Figure 2 shows the relationship between total scar burden, scan quality and PVE. It is clear that there is a significant relationship between PVE and scar burden (R^2^ for both vein pairs 0.632 (linear regression) and 0.818 (one phase decay regression)), but that scar burden is not the only determinant of PVE. Likewise, there is a significant relationship between PVE and overall scan quality (R^2^ for both vein pairs 0.302), but a high degree of PVE can still be detected on relatively poor quality scans.


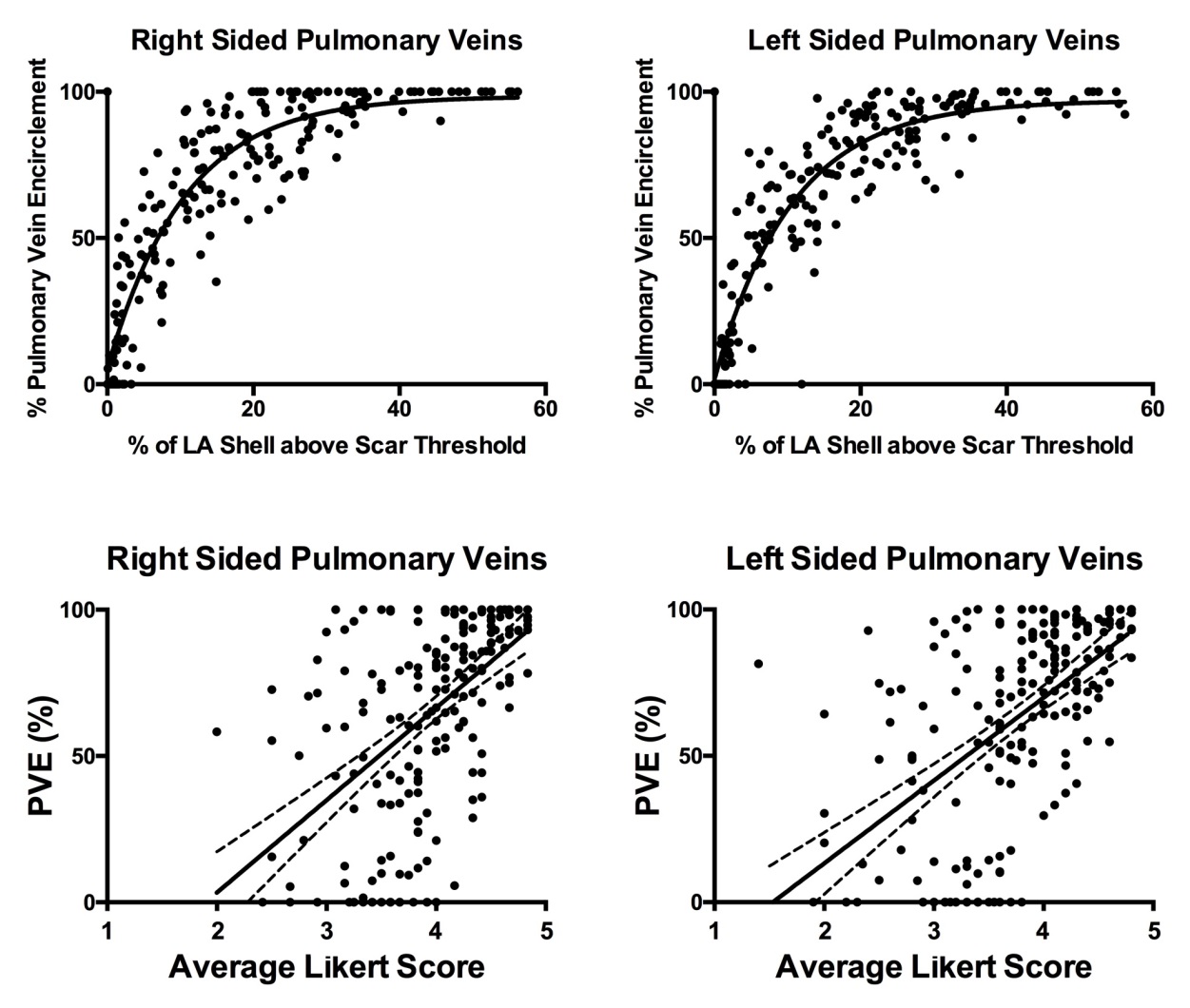


Additional file 1: Figure S2. The dependence of pulmonary vein encirclement (PVE) upon scan parameters.

(Top row) Percentage PVE against the proportion of the whole LA scar that is above the threshold of 3.3 standard deviations above the blood pool mean. Note the increase in PVE with increase in total scar burden, but that in some cases complete PVE may be observed in the presence of low (<20%) total scar burden, whilst in others a much larger proportion of the shell may be ascribed to scar status without complete PVE. (Bottom row) Percentage PVE against scan quality, expressed in terms of overall average Likert Score, across the four criteria. Again, there is a general increase in % PVE with improved scan quality, but a wide range of degree of PVE is observed with both high and low quality scans.

## References

1. Khurram IM, Beinart R, Zipunnikov V, Dewire J, Yarmohammadi H, Sasaki T, et al. Magnetic resonance image intensity ratio, a normalized measure to enable interpatient comparability of left atrial fibrosis. Heart Rhythm. Elsevier; 2014;11:85–92.

2. Groarke JD, Waller AH, Vita TS, Michaud GF, Di Carli MF, Blankstein R, et al. Feasibility study of electrocardiographic and respiratory gated, gadolinium enhanced magnetic resonance angiography of pulmonary veins and the impact of heart rate and rhythm on study quality. J. Cardiovasc. Magn. Reson. 2014;16:43.

3. Tobon-Gomez C, Zuluaga MA, Chubb H, Williams SE, Butakoff C, Karim R, et al. Standardised unfold map of the left atrium: regional definition for multimodal image analysis. J. Cardiovasc. Magn. Reson. 2015;17:P41.

4. Tobon-Gomez C, Geers A, Peters J, Weese J, Pinto K, Karim R, et al. Benchmark for algorithms segmenting the left atrium from 3D CT and MRI datasets. IEEE Trans. Med. Imaging. 2015;62:1–1.

5. Durrleman S, Prastawa M, Charon N, Korenberg JR, Joshi S, Gerig G, et al. Morphometry of anatomical shape complexes with dense deformations and sparse parameters. Neuroimage. 2014;101:35–49.

6. Nuñez Garcia M, Tobon-Gomez C, Rhode K, Bijnens B, Camara O, Butakoff C. Quantification of Gaps in Ablation Lesions Around the Pulmonary Veins in Delayed Enhancement MRI. Springer International Publishing; 2015. p. 215–22.
